# Supplementary material for: Genome-wide quantification of the effects of DNA methylation on human gene regulation
Source: eLife. 2018 Dec 21;7:e37513. doi: 10.7554/eLife.37513 (PMC6303109; doi:10.7554/eLife.37513)
Supplement: Transparent reporting form [file elife-37513-transrepform.docx]

***eLife’s* transparent reporting form**

We encourage authors to provide detailed information *within their submission* to facilitate the interpretation and replication of experiments. Authors can upload supporting documentation to indicate the use of appropriate reporting guidelines for health-related research (see [EQUATOR Network](http://www.equator-network.org/%20)), life science research (see the [BioSharing Information Resource](https://biosharing.org/)), or the [ARRIVE guidelines](http://www.plosbiology.org/article/info:doi/10.1371/journal.pbio.1000412) for reporting work involving animal research. Where applicable, authors should refer to any relevant reporting standards documents in this form.

If you have any questions, please consult our Journal Policies and/or contact us: [editorial@elifesciences.org](mailto:editorial@elifesciences.org).

**Sample-size estimation**

- You should state whether an appropriate sample size was computed when the study was being designed
- You should state the statistical method of sample size computation and any required assumptions
- If no explicit power analysis was used, you should describe how you decided what sample (replicate) size (number) to use

Please outline where this information can be found within the submission (e.g., sections or figure legends), or explain why this information doesn’t apply to your submission:

Information about sample size selection and justification can be found at lines 116-121.

**Replicates**

- You should report how often each experiment was performed
- You should include a definition of biological versus technical replication
- The data obtained should be provided and sufficient information should be provided to indicate the number of independent biological and/or technical replicates
- If you encountered any outliers, you should describe how these were handled
- Criteria for exclusion/inclusion of data should be clearly stated
- High-throughput sequence data should be uploaded before submission, with a private link for reviewers provided (these are available from both GEO and ArrayExpress)

Please outline where this information can be found within the submission (e.g., sections or figure legends), or explain why this information doesn’t apply to your submission:

Information about replicates can be found at lines 116-121. We excluded one replicate because of low sequencing depth, this is described at lines 462-465. High throughput sequencing data has been uploaded through NCBI’s SRA (accession number = SRP120556).

**Statistical reporting**

- Statistical analysis methods should be described and justified
- Raw data should be presented in figures whenever informative to do so (typically when N per group is less than 10)
- For each experiment, you should identify the statistical tests used, exact values of N, definitions of center, methods of multiple test correction, and dispersion and precision measures (e.g., mean, median, SD, SEM, confidence intervals; and, for the major substantive results, a measure of effect size (e.g., Pearson's r, Cohen's d)
- Report exact p-values wherever possible alongside the summary statistics and 95% confidence intervals. These should be reported for all key questions and not only when the p-value is less than 0.05.

Please outline where this information can be found within the submission (e.g., sections or figure legends), or explain why this information doesn’t apply to your submission:

We report exact p-values wherever possible. In a few cases, we report the minimum p-value provided by the statistical program we used. In the results, we state p-values, effect sizes, and the test that was used to generate the result in the following locations:

Test for enhancer activity (linear model) – described at lines 145-146, p-values provided in Table S3

Test for MD enhancer activity (linear model) – described at lines 146-149, p-values provided in Table S4

Test for agreement between low throughput methods and mSTARR-seq (linear model) – described at lines 160-164

Test for enrichment of mSTARR-seq identified enhancers in external enhancer annotation datasets (Fisher’s exact test) – described at lines 168-171

Test to ask whether effect sizes for mSTARR-seq enhancers are larger in regions annotated by ENCODE as enhancers (linear model) – described at lines 173-175

Test to ask whether MD enhancers have higher CpG density than non MD enhancers (Wilcoxon test) and whether CpG density predicts MD activity – described at lines 187-192

Test to see if MD enhancers could be predicted from various features (Random forest) – described at lines 200-203

Test for agreement between MD effect size and effect sizes for methylation sensitivity estimated from previous experiments (linear model) – described at lines 220-225

Test for enrichment of particular TF families in MD enhancers (hypergeometric test) – described at lines 226-229 (full results provided in Table S8)

Test for enrichment of MD enhancers in regions where DNA methylation variation predicts expression variation in the endogenous genome (Fisher’s exact test) – described at lines 253-259

(For large datasets, or papers with a very large number of statistical tests, you may upload a single table file with tests, Ns, etc., with reference to sections in the manuscript.)

**Group allocation**

- Indicate how samples were allocated into experimental groups (in the case of clinical studies, please specify allocation to treatment method); if randomization was used, please also state if restricted randomization was applied
- Indicate if masking was used during group allocation, data collection and/or data analysis

Please outline where this information can be found within the submission (e.g., sections or figure legends), or explain why this information doesn’t apply to your submission:

This is an experimental laboratory study, samples were treated identically except for a chemical manipulation performed on half the samples. Masking was not used during group allocation.

**Additional data files (“source data”)**

- We encourage you to upload relevant additional data files, such as numerical data that are represented as a graph in a figure, or as a summary table
- Where provided, these should be in the most useful format, and they can be uploaded as “Source data” files linked to a main figure or table
- Include model definition files including the full list of parameters used
- Include code used for data analysis (e.g., R, MatLab)
- Avoid stating that data files are “available upon request”

Please indicate the figures or tables for which source data files have been provided:

Source data are provided for:

Figure 1E in Table S1

Figure 2C in Table S5

Figure 3A in Table S4

Figure 3F in Table S6

Figure 4A-B in Table S7-S8

All raw data are available through NCBI’s SRA (accession number = SRP120556).
